# Bakuchiol Enhances 5-Fluorouracil Efficacy in Colorectal Cancer Cells via a ROS-Dependent Mechanism Involving Mitochondrial Dysfunction and Apoptosis

**DOI:** 10.3390/ijms27135894

**Published:** 2026-06-30

**Authors:** Dominika Radomska, Olga Szewczyk-Roszczenko, Magda Chalecka, Arkadiusz Surazynski, Anna Szymanowska, Krzysztof Bielawski, Robert Czarnomysy

**Affiliations:** 1 Department of Synthesis and Technology of Drugs, Medical University of Bialystok, Kilinskiego 1, 15-089 Bialystok, Poland; donius2410@gmail.com (D.R.); olga.szewczyk-roszczenko@umb.edu.pl (O.S.-R.); anna.szymanowska@umb.edu.pl (A.S.); krzysztof.bielawski@umb.edu.pl (K.B.); 2 Department of Medicinal Chemistry, Medical University of Bialystok, Mickiewicza 2D, 15-222 Bialystok, Poland; magda.chalecka@umb.edu.pl (M.C.); arkadiusz.surazynski@umb.edu.pl (A.S.)

**Keywords:** colorectal cancer, anticancer activity, natural compounds, bakuchiol, 5-FU, combination therapy, cancer therapy, cancer co-treatment, chemotherapy

## Abstract

Resistance to 5-fluorouracil (5-FU) remains a major limitation in colorectal cancer therapy, prompting the development of combination strategies aimed at improving its efficacy. Bakuchiol (BAK), a natural compound with reported antioxidant and pro-oxidant properties, may modulate redox balance and enhance chemotherapy response. This study compared the effects of 5-FU and BAK, applied as monotherapies and in combination, in DLD-1 and HT-29 colorectal cancer cells. Cytotoxicity assays showed that co-treatment significantly reduced the IC_50_ of 5-FU, particularly in DLD-1 cells, and revealed an enhanced anticancer effect of the combination treatment compared with either monotherapy. Flow cytometric analyses demonstrated enhanced apoptosis via extrinsic and intrinsic pathways, including increased caspase 8 activity, loss of mitochondrial membrane potential (ΔΨ_m_), activation of caspase 9, and subsequent activation of caspases 3/7. These effects were associated with a pronounced redox imbalance, reflected by increased intracellular reactive oxygen species (ROS) levels, suggesting a central role of oxidative stress in mediating cytotoxicity. Antioxidant pre-treatment attenuated ROS accumulation and reduced apoptosis, confirming a causal relationship. Additionally, autophagy was induced selectively in DLD-1 cells, indicating cell-line-specific differences in redox adaptation. Taken together, BAK enhances 5-FU efficacy through ROS-dependent activation of mitochondrial and caspase-dependent pathways, with stronger effects observed in DLD-1 cells.

## 1. Introduction

For many years, clinicians worldwide have faced the challenge of cancer. This issue remains highly relevant, as the number of newly diagnosed cases continues to rise and, despite the availability of modern therapeutic approaches, cancer-related mortality remains high. Colorectal cancer (CRC) is one of the most common malignancies globally. According to the latest GLOBOCAN 2022 estimates, approximately 1.9 million new CRC cases and over 900,000 deaths were reported worldwide, highlighting the substantial global burden of this disease [1]. Moreover, epidemiological projections indicate a further increase in CRC incidence in the coming years. Additionally, the American Cancer Society estimates that in the United States alone, 154,270 new CRC cases will be diagnosed in 2025 [2]. These data are alarming and underscore the urgent need to develop new therapeutic strategies that could effectively reduce cancer-related mortality or enable accurate diagnosis at very early stages of the disease.

**Figure 1:**
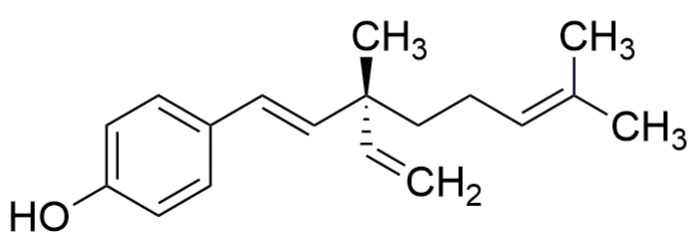
Chemical structure of bakuchiol (4-[(1*E*,3*S*)-3-ethenyl-3,7-dimethyl-1,6-octadien-1-yl]phenol).

Synthetic drugs often fail to fully address the challenges of modern oncology; therefore, there is a continuous need to identify new chemotherapeutic agents, including those derived from natural sources. Numerous natural compounds with cytostatic activity are already used in clinical practice, such as Vinca alkaloids (vinblastine, vincristine), paclitaxel, bleomycin, and anthracycline antibiotics (daunorubicin, doxorubicin [3]. Among such naturally derived compounds, bakuchiol (BAK, Figure 1) has recently attracted increasing attention. Bakuchiol (BAK) is a botanical chemical compound (meroterpene) isolated from the edible seeds of the Indian plant species *Cullen corylifolium* L. Medik (formerly *Psoralea corylifolia* L., *Fabaceae* family), widely used in cosmetology and dermatology [4,5,6]. The available literature also highlights its hypoglycemic, cardioprotective and hepatoprotective and anticancer properties [4,5,6]. In oncology studies, the compound has exhibited cytotoxic effects against many types of cancer, including, breast cancer [7,8], prostate [9], lung [8,10] and gastric [11,12] cancers.

Therapeutic management of CRC includes multiple approaches, such as oncologic surgery, radiotherapy, immunotherapy, targeted therapy, and chemotherapy. Among the agents used in CRC treatment are pembrolizumab, bevacizumab, cetuximab, irinotecan, and 5-fluorouracil (5-FU), the latter being widely applied as a first-line therapy [13,14]. However, its clinical utility is significantly limited by the rapid development of resistance. It has been reported that no more than 15% of patients with advanced CRC respond positively to 5-FU monotherapy. Encouragingly, co-treatment with other compounds exhibiting anticancer activity can increase the response rate to as much as 50% [14,15]. Therefore, combination therapy represents a promising strategy, as it may reduce the required drug doses—thereby limiting side effects—while simultaneously enhancing therapeutic efficacy [16].

Given these properties and considering the limitations associated with 5-FU monotherapy, the aim of the present study was to evaluate the anticancer potential of BAK used alone and in combination with 5-FU in colorectal cancer models (DLD-1 and HT-29). Particular attention was paid to determining whether BAK can enhance the efficacy of 5-FU and act as a sensitizing agent by modulating key cellular processes, including oxidative stress, apoptosis, and autophagy.

## 2. Results

### 2.1. BAK and Its Combination Therapy with 5-FU Reduces Colorectal Cancer Cell Viability (MTT)

**Figure 2:**
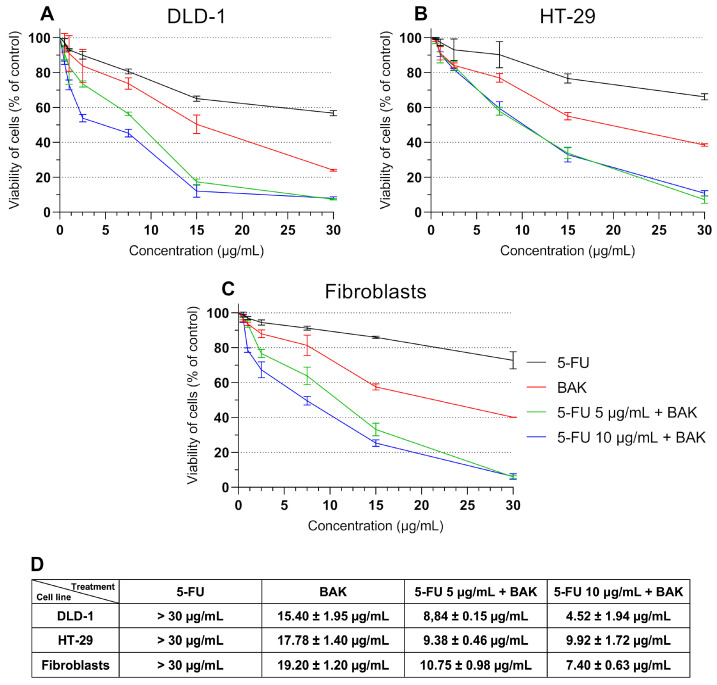
Viability of DLD-1 (**A**), HT-29 (**B**) colorectal cancer cells and fibroblast cells (**C**) determined by MTT assay after 24 h incubation with 5-FU, BAK, and 5-FU + BAK at different concentrations (0.5–30 µg/mL) and obtained IC_50_ values (**D**). Data are presented as a percentage of control (mean ± SD, *n* = 3).

The in vitro cytotoxicity of 5-FU, BAK, and their combination therapy against human colorectal cancer cells (DLD-1 and HT-29) was evaluated after 24 h at various concentrations (0.5–30 µg/mL). The results are shown in Figure 2. The IC_50_ of 5-FU exceeded the tested concentration range in both colorectal cancer cell lines, indicating limited efficacy of the monotherapy under these conditions. In contrast, treatment with BAK alone resulted in moderate cytotoxicity. Notably, the combination of 5-FU (5 and 10 µg/mL) with BAK markedly enhanced the antiproliferative effect, leading to a substantial reduction in IC_50_ values to 8.84 ± 0.15 and 4.52 ± 1.94 µg/mL in DLD-1 cells, and to 12.15 ± 0.36 and 11.83 ± 0.15 µg/mL in HT-29 cells. A similar effect was observed in fibroblasts, where the combination treatment also reduced IC_50_ values (10.75 ± 0.98 and 7.40 ± 0.63 µg/mL for 5 and 10 µg/mL 5-FU with BAK, respectively), indicating that the combined therapy is not entirely selective and exhibits cytotoxicity toward non-cancer cells. However, it is important to note that the cytotoxic effect of the combination was more pronounced in DLD-1 cells, suggesting a degree of preferential activity against cancer cells.

Building on the cytotoxicity results, we further evaluated the selectivity and interaction profile of the combined treatment (Table 1). The selectivity index (SI), calculated as the ratio of IC_50_ in fibroblasts to that in cancer cells, indicated only moderate preferential activity toward malignant cells. In DLD-1 cells, SI values were 1.22 and 1.64 for combinations with 5 and 10 µg/mL 5-FU, respectively, suggesting improved selectivity at the higher dose. In contrast, HT-29 cells exhibited lower selectivity, particularly at 10 µg/mL (SI = 0.75), indicating comparable or even greater sensitivity of fibroblasts under these conditions. To further assess the interaction between BAK and 5-FU, a combination effect index was calculated based on IC_50_ reduction relative to BAK alone. The combination treatment demonstrated a marked enhancement of cytotoxic activity compared with BAK monotherapy, especially in DLD-1 cells, where synergy indices reached 1.74 and 3.41 for 5 and 10 µg/mL 5-FU, respectively. In HT-29 cells, the effect was less pronounced but suggested a positive interaction between both compounds (1.79–1.90). Taken together, these findings confirm that the combined BAK and 5-FU treatment enhances cytotoxic efficacy compared to monotherapies, with the most favorable therapeutic profile observed in DLD-1 cells, where increased potency is accompanied by improved selectivity.

**Figure 3:**
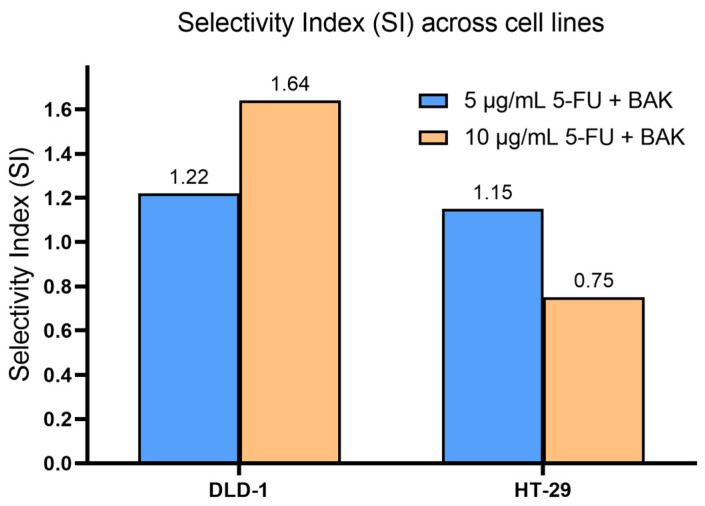
Selectivity index (SI) of combined BAK and 5-FU treatment in colorectal cancer cell lines. The selectivity index (SI) was calculated as the ratio of IC_50_ values in fibroblasts to those in cancer cells. Higher SI values indicate greater preferential cytotoxicity toward cancer cells.

The selectivity index (SI) was further visualized using a bar plot to compare the relative therapeutic window across cell lines (Figure 3). The analysis demonstrated that DLD-1 cells exhibited higher selectivity, particularly for the combination with 10 µg/mL 5-FU, whereas HT-29 cells showed reduced selectivity under the same conditions. Notably, the decrease in SI in HT-29 at higher drug concentrations indicates a loss of preferential toxicity toward cancer cells.

### 2.2. BAK and Its Combination Therapy with 5-FU Activates the Extrinsic and Intrinsic Apoptotic Pathway

**Figure 4:**
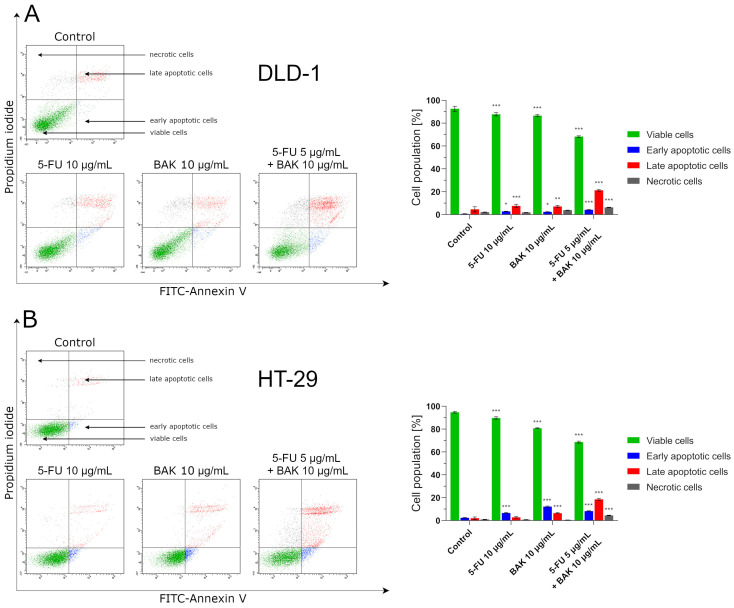
Flow cytometry analysis of apoptosis induction in DLD-1 (**A**) and HT-29 (**B**) colorectal cancer cells after 24 h incubation with 5-FU, BAK, and 5-FU + BAK (monotherapy: 10 µg/mL, combined treatment: 5 + 10 µg/mL) and staining with Annexin V-FITC/PI. Data are presented as mean ± SD (*n* = 3). * *p* < 0.05 vs. control group, ** *p* < 0.01 vs. control group, *** *p* < 0.001 vs. control group.

Flow cytometric analysis performed after 24 h incubation demonstrated that the cytotoxic effects observed in the MTT assay are associated with apoptosis induction, particularly under combination treatment conditions (Figure 4). In both DLD-1 and HT-29 colorectal cancer cell lines, co-treatment with 5-FU (5 µg/mL) and BAK (10 µg/mL) significantly increased the percentage of apoptotic cells (early + late apoptosis) compared to monotherapies. In DLD-1 cells, BAK alone induced apoptosis in 9.6 ± 0.9% of cells, whereas the combination treatment increased this fraction to 25.5 ± 1.0%, corresponding to an approximately 2.66-fold increase (+166%) relative to BAK monotherapy. In contrast, 5-FU alone triggered only a weak apoptotic response (5.2 ± 0.6%). In HT-29 cells, BAK alone resulted in 16.7 ± 1.7% apoptotic cells, while the combination increased this value to 26.8 ± 1.3%, representing a 1.60-fold increase (+60%). Notably, 5-FU monotherapy induced a slightly higher level of apoptosis in HT-29 cells (9.5 ± 0.9%) than in DLD-1 cells. Importantly, although the final percentage of apoptotic cells after combination treatment was comparable between both cell lines, the relative enhancement of apoptosis was markedly greater in DLD-1 cells. This indicates a stronger potentiation effect of BAK on 5-FU-induced apoptosis in this model.

These findings are consistent with the MTT assay results, where a synergistic interaction between BAK and 5-FU was observed in DLD-1 cells (SI = 1.65), while HT-29 cells exhibited a non-synergistic response (SI = 0.75). The substantially higher fold increase in apoptosis in DLD-1 cells supports the conclusion that the combination treatment exerts a more pronounced pro-apoptotic and cytotoxic effect in this cell line.

**Figure 5:**
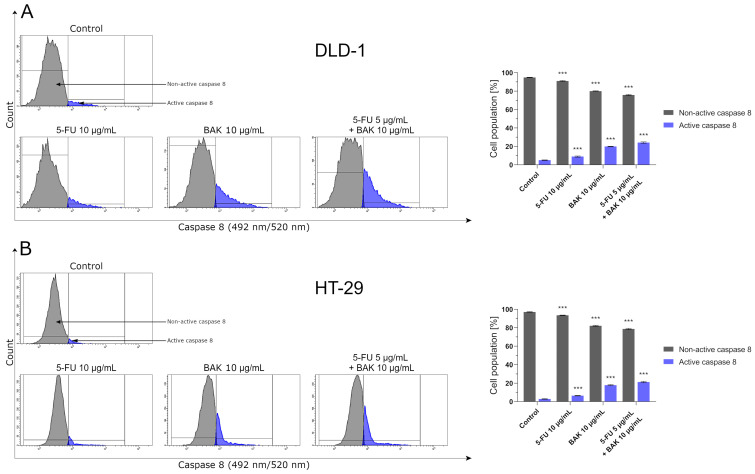
Flow cytometry analysis of caspase 8 activity in DLD-1 (**A**) and HT-29 (**B**) colorectal cancer cells after 24 h incubation with 5-FU, BAK, and 5-FU + BAK (monotherapy: 10 µg/mL, combined treatment: 5 + 10 µg/mL). Data are presented as mean ± SD (*n* = 3).*** *p* < 0.001 vs. control group.

Since activation of caspase 8 is a key event in the initiation of apoptosis via the extrinsic pathway [17], we evaluated its activity in DLD-1 and HT-29 colorectal cancer cells following 24 h treatment with 5-FU, BAK, and their combination. Flow cytometric analysis revealed that both monotherapies induced activation of caspase 8; however, the effect was more pronounced after BAK treatment than after 5-FU alone (Figure 5). In DLD-1 cells, BAK increased the fraction of cells with active caspase 8 to 19.6 ± 1.1%, compared to 9.0 ± 0.6% observed for 5-FU. Similarly, in HT-29 cells, BAK induced caspase 8 activation in 17.9 ± 0.6% of cells, whereas 5-FU resulted in 6.5 ± 0.3%. Importantly, the combination of 5-FU (5 µg/mL) with BAK (10 µg/mL) further enhanced caspase 8 activation in both cell lines. In DLD-1 cells, the percentage of cells with active caspase 8 increased to 24.2 ± 0.7%, corresponding to an approximately 1.23-fold increase (+23%) compared to BAK monotherapy. In HT-29 cells, the combination treatment resulted in 21.3 ± 0.8% active caspase 8-positive cells, representing a 1.19-fold increase (+19%). Although the absolute differences between combination treatment and BAK alone were moderate, the relative enhancement of caspase 8 activation was slightly more pronounced in DLD-1 cells. This trend is consistent with the Annexin V/PI apoptosis assay, where a stronger pro-apoptotic effect of the combination treatment was observed in DLD-1 compared to HT-29 cells.

**Figure 6:**
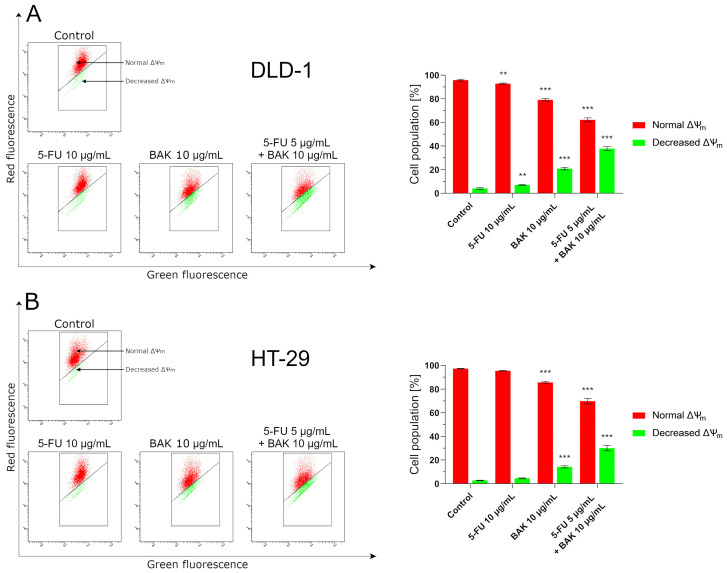
Flow cytometry analysis of mitochondrial membrane potential changes (MMP, ΔΨ_m_) in DLD-1 (**A**) and HT-29 (**B**) colorectal cancer cells after 24 h incubation with 5-FU, BAK, and 5-FU + BAK (monotherapy: 10 µg/mL, combined treatment: 5 + 10 µg/mL). Data are presented as mean ± SD (*n* = 3). ** *p* < 0.01 vs. control group, *** *p* < 0.001 vs. control group.

To further investigate early events associated with apoptosis induction, changes in mitochondrial membrane potential (ΔΨ_m_) were analyzed using JC-1 staining (Figure 6). This parameter reflects one of the earliest steps of the intrinsic apoptotic pathway and therefore provides important insight into the initiation of cell death [18]. Treatment with 5-FU alone had only a marginal impact on mitochondrial integrity, as evidenced by a low percentage of cells with decreased ΔΨ_m_ in both DLD-1 (7.1 ± 0.5%) and HT-29 (4.5 ± 0.4%). In contrast, BAK alone induced a clear increase in mitochondrial depolarization, particularly in DLD-1 cells (20.9 ± 1.1%), compared to HT-29 (14.2 ± 0.9%), indicating a higher baseline sensitivity of this cell line to BAK. Notably, the combined treatment further intensified this effect, leading to a substantial accumulation of cells with disrupted mitochondrial potential. In DLD-1 cells, the fraction of depolarized mitochondria reached 37.8 ± 1.7%, whereas in HT-29 cells it increased to 30.1 ± 2.3%. Although both cell lines responded to the combined treatment, the overall level of mitochondrial dysfunction remained consistently higher in DLD-1 cells.

Importantly, when considered together with the Annexin V/PI results, these findings suggest a coherent sequence of events, where the combined treatment more effectively initiates early mitochondrial alterations, subsequently leading to enhanced apoptosis. This effect is particularly evident in DLD-1 cells, where both the early (ΔΨ_m_ loss) and later (phosphatidylserine externalization) markers of apoptosis are more strongly pronounced.

**Figure 7:**
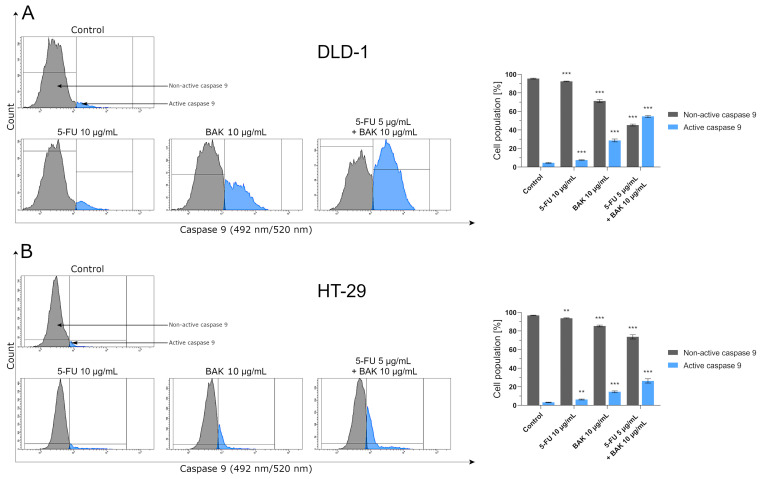
Flow cytometry analysis of caspase 9 activity in DLD-1 (**A**) and HT-29 (**B**) colorectal cancer cells after 24 h incubation with 5-FU, BAK, and 5-FU + BAK (monotherapy: 10 µg/mL, combined treatment: 5 + 10 µg/mL). Data are presented as mean ± SD (*n* = 3). ** *p* < 0.01 vs. control group, *** *p* < 0.001 vs. control group.

Given the previously observed disruption of mitochondrial membrane potential, we next examined whether these changes were accompanied by activation of caspase 9, a central component of the intrinsic apoptotic pathway [18,19]. In line with earlier observations, 5-FU alone exerted only a minor effect on caspase 9 activation in both cell lines (Figure 7). A more evident response was detected following BAK treatment, particularly in DLD-1 cells, where the proportion of cells with active caspase 9 reached 28.4 ± 1.6%, compared to 14.6 ± 1.0% in HT-29 cells. The most pronounced changes, however, were associated with the combined treatment. In DLD-1 cells, caspase 9 activation increased sharply, with more than half of the cell population (54.7 ± 2.3%) exhibiting the active form of the enzyme. In contrast, HT-29 cells displayed a considerably lower response under the same conditions (29.3 ± 2.2%). Although both models responded to the combined treatment, the magnitude of the effect clearly differed. The markedly higher proportion of caspase 9-positive cells in DLD-1 indicates a more efficient propagation of the mitochondrial apoptotic signal in this cell line. This observation is in agreement with the stronger mitochondrial depolarization detected earlier and suggests that, in DLD-1 cells, the intrinsic pathway is not only initiated but also more effectively amplified.

**Figure 8:**
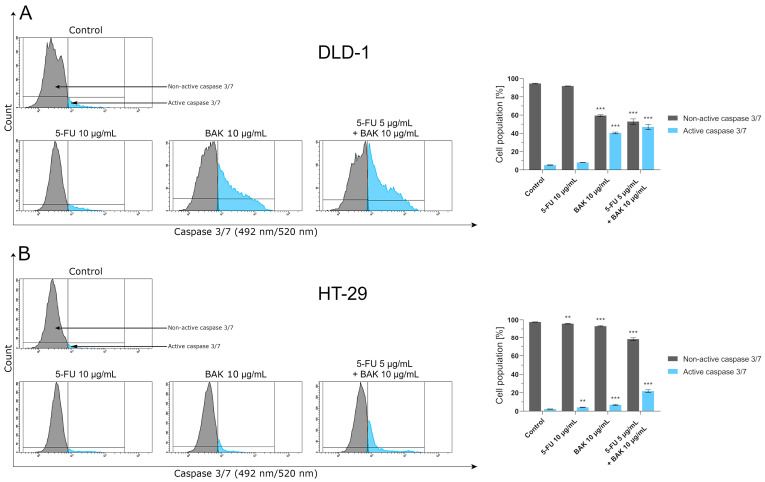
Flow cytometry analysis of caspase 3/7 activity in DLD-1 (**A**) and HT-29 (**B**) colorectal cancer cells after 24 h incubation with 5-FU, BAK, and 5-FU + BAK (monotherapy: 10 µg/mL, combined treatment: 5 + 10 µg/mL). Data are presented as mean ± SD (*n* = 3). ** *p* < 0.01 vs. control group, *** *p* < 0.001 vs. control group.

To capture the final stage of apoptosis, we examined the activation of executioner caspases 3 and 7, which integrate signals from both the extrinsic and intrinsic pathways and ultimately determine cell fate [20,21]. The pattern of response differed noticeably between the two cell lines (Figure 8). In HT-29 cells, 5-FU alone induced a modest but significant increase in caspase 3/7 activity, whereas BAK and especially the combined treatment further enhanced this effect, reaching 6.9 ± 0.6% and 21.9 ± 1.4% of caspase 3/7-positive cells, respectively. In contrast, DLD-1 cells displayed a markedly stronger activation of executioner caspases. Even BAK monotherapy resulted in a substantial proportion of cells entering the execution phase (40.5 ± 1.0%), which further increased to 47.1 ± 2.9% following combination treatment. Thus, nearly half of the DLD-1 cell population exhibited active caspase 3/7 under co-treatment conditions.

This pronounced difference between the cell lines highlights a key aspect of their response: while HT-29 cells show a gradual increase in apoptotic signaling, DLD-1 cells undergo a far more extensive transition into the terminal phase of apoptosis. Notably, the magnitude of caspase 3/7 activation in DLD-1 cells clearly exceeds that observed at earlier stages of the apoptotic cascade, indicating efficient propagation and amplification of the death signal.

**Figure 9:**
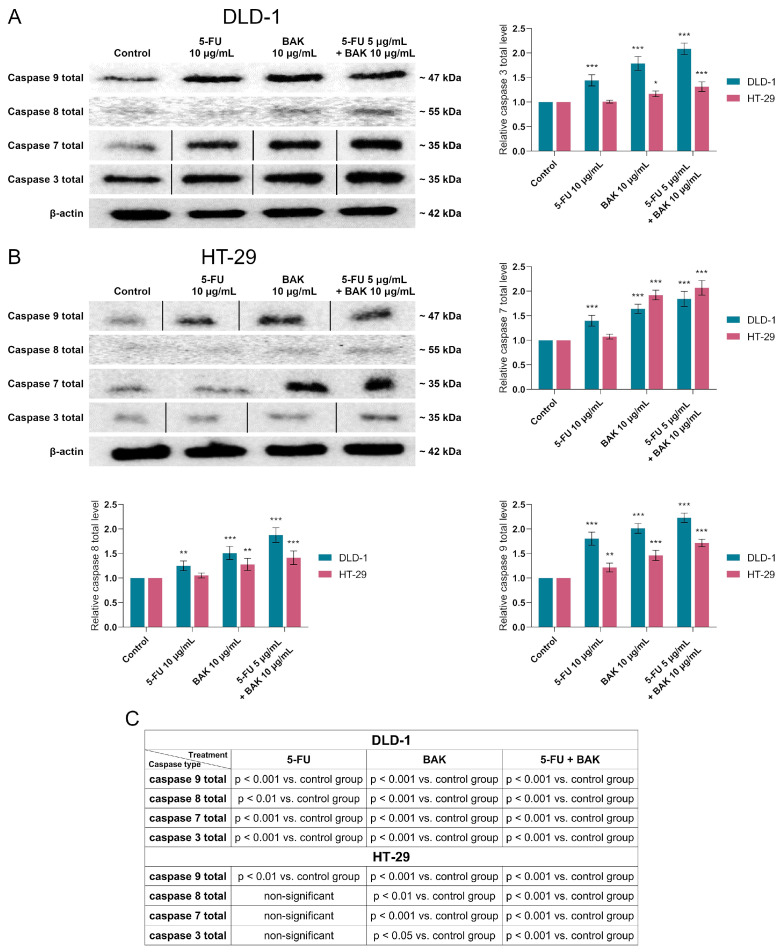
Western blot analysis of total caspase 3, 7, 8, and 9 expression in colorectal cancer cells. DLD-1 (**A**) and HT-29 (**B**) cells were treated for 24 h with 5-FU (10 µg/mL), bakuchiol (10 µg/mL), or their combination (5-FU 5 µg/mL + bakuchiol 10 µg/mL). Panel (**C**) summarizes the statistical significance of changes in caspase expression relative to the control group. Protein levels were normalized to β-actin and expressed relative to control. Lanes shown in the figure were rearranged from the same membrane to present the treatment groups in a consistent order across all analyzed proteins; splicing is indicated by vertical lines. No bands were altered or selectively modified. Each protein was analyzed on separate membranes. Data are presented as mean ± SD (*n* = 3). * *p* < 0.05 vs. control group, ** *p* < 0.01 vs. control group, *** *p* < 0.001 vs. control group. Full, uncropped blots are provided in the Supplementary Materials.

To validate the flow cytometry findings at the protein level, the total expression of initiator and executioner caspases (caspase 3, 7, 8, and 9) was assessed by Western blot after 24 h of treatment (Figure 9; for original membrane images from Western blot, see Supplementary Materials). The obtained results closely reflected the patterns observed in the cytometric assays, while also providing additional insight into the regulation of apoptotic machinery at the expression level. Notably, the response to 5-FU alone differed substantially between the two cell lines. In HT-29 cells, this treatment did not significantly alter the expression of caspase 3 and 8, whereas in DLD-1 cells, a clear upregulation was already evident (1.44 ± 0.11-fold for caspase 3 and 1.25 ± 0.10-fold for caspase 8), indicating an earlier and more pronounced engagement of apoptotic signaling. Treatment with BAK alone led to a consistent increase in the expression of all analyzed caspases in both models. However, the extent and pattern of these changes differed. In DLD-1 cells, the upregulation was balanced across both initiator and executioner caspases (1.79 ± 0.14, 1.64 ± 0.09, 1.81 ± 0.13, and 2.01 ± 0.10-fold for caspase 3, 7, 8, and 9, respectively), suggesting coordinated activation of the apoptotic cascade. In HT-29 cells, although increases were also observed, they were generally less uniform and, in the case of some caspases, less pronounced in relation to the overall apoptotic outcome. The most distinctive effect was associated with the combined treatment. Co-administration of 5-FU and BAK resulted in the highest expression levels of all examined caspases in both cell lines, exceeding the effects of individual treatments. Importantly, in DLD-1 cells, this increase corresponded with the previously observed strong activation of caspases (flow cytometry) and high levels of apoptosis, indicating efficient propagation of the apoptotic signal from initiation to execution stages. When considered together with earlier results—mitochondrial depolarization, activation of caspase 9 and 8, and finally caspase 3/7 activity—the Western blot data confirm a consistent and amplified apoptotic response in DLD-1 cells. In contrast, although HT-29 cells also respond to the combined treatment, the overall effect remains less pronounced, particularly at the level of executioner caspase activation.

BAK was applied as monotherapy (10 µg/mL) and as a combined treatment (5 + 10 µg/mL). For the experiment, equal amounts (30 µg/lane) of protein lysates were used (for original membrane images from Western blot, see Supplementary Materials). The intensity of band staining was quantified by densitometric analysis. The obtained results are presented as mean values ± SD obtained from three independent experiments (*n* = 3) done in triplicate. (**C**) presents a table with *p*-values for both cell lines and each caspase type compared to the control group.

### 2.3. BAK and Its Combination Therapy with 5-FU Selectively Activates Autophagy in DLD-1 Cells

**Figure 10:**
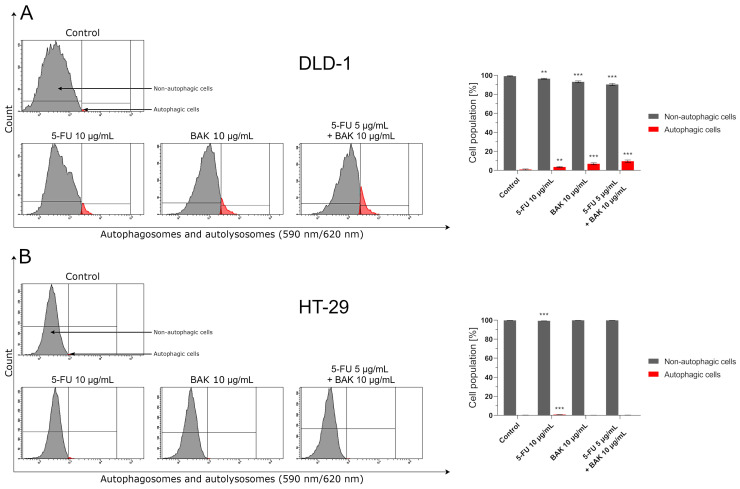
Flow cytometry analysis of autophagy induction (detection of autophagosomes and autolysosomes using the Autophagy Probe, Red) in DLD-1 (**A**) and HT-29 (**B**) colorectal cancer cells after 24 h incubation with 5-FU, BAK, and 5-FU + BAK (monotherapy: 10 µg/mL, combined treatment: 5 + 10 µg/mL). Data are presented as mean ± SD (*n* = 3).** *p* < 0.01 vs. control group, *** *p* < 0.001 vs. control group.

Given the pronounced mitochondrial dysfunction (ΔΨ_m_ loss) and activation of caspase-dependent apoptosis observed previously, we investigated whether these stress signals are accompanied by autophagy induction. At the molecular level, autophagy is commonly triggered by mitochondrial damage and oxidative stress, serving to remove dysfunctional organelles (e.g., via mitophagy) and maintain cellular homeostasis. However, under excessive stress conditions, it may also contribute to the progression of cell death [22,23]. In this context, the analysis of autophagy revealed a distinctly asymmetric response between the two colorectal cancer models (Figure 10). HT-29 cells remained largely unresponsive, with autophagy levels comparable to control conditions, except for a slight increase following 5-FU treatment (0.8 ± 0.1% vs. 0.2 ± 0.1%). In contrast, DLD-1 cells exhibited a clear and treatment-dependent induction of autophagy, increasing from 3.6 ± 0.5% (5-FU) to 6.9 ± 1.0% (BAK) and reaching 9.7 ± 1.2% under combination treatment. Although the magnitude of autophagy was lower than that observed for apoptosis, its selective activation in DLD-1 cells is notable. In the context of the strong apoptotic response previously described, autophagy appears to accompany—rather than counteract—cell death signaling.

The absence of a comparable response in HT-29 cells further highlights their limited sensitivity, whereas DLD-1 cells engage a broader, more integrated stress response involving both apoptotic and autophagic pathways, which may underlie their higher susceptibility to the combined treatment.

### 2.4. BAK and Its Combination Therapy with 5-FU Elevate Intracellular ROS Levels

**Figure 11:**
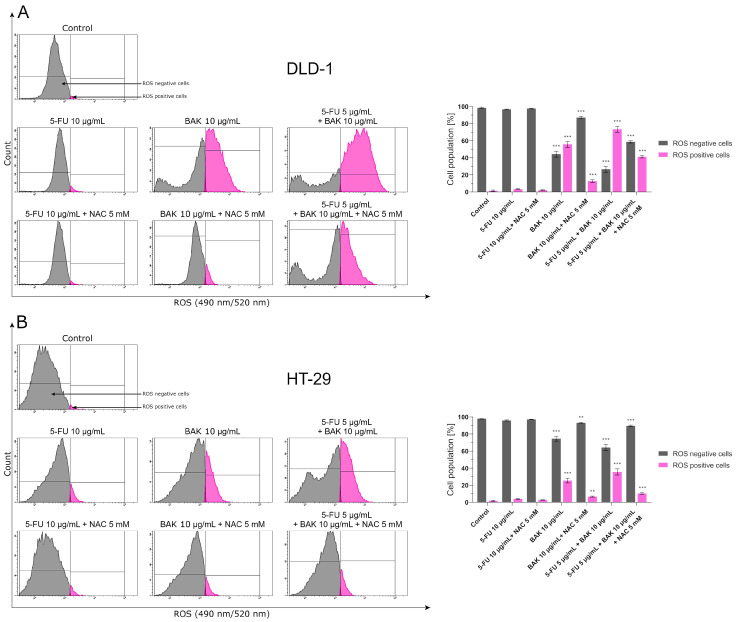
Flow cytometry analysis of intracellular ROS levels in DLD-1 (**A**) and HT-29 (**B**) colorectal cancer cells after pre-treatment with or without 5 mM NAC for 1 h followed by 24 h incubation with 5-FU, BAK, and 5-FU + BAK (monotherapy: 10 µg/mL, combined treatment: 5 + 10 µg/mL). Data are presented as mean ± SD (*n* = 3). ** *p* < 0.01 vs. control group, *** *p* < 0.001 vs. control group.

Considering the previously observed mitochondrial dysfunction and induction of autophagy in DLD-1 cells, we further examined whether oxidative stress may act as an upstream trigger of these events (Figure 11). At the cellular level, excessive ROS accumulation is known to disrupt mitochondrial integrity, promote ΔΨ_m_ loss, and activate both apoptotic and autophagic pathways [23]. Flow cytometric analysis revealed that 5-FU alone had a minimal effect on ROS generation in both cell lines. In contrast, BAK treatment led to a substantial increase in ROS-positive cells, which was further intensified under combination treatment. Notably, this effect was markedly stronger in DLD-1 cells, where ROS levels reached 55.8 ± 3.5% (BAK) and 73.4 ± 3.4% (5-FU + BAK), compared to 25.5 ± 2.8% and 35.6 ± 3.2% in HT-29 cells, respectively. The involvement of oxidative stress in the observed cellular responses was confirmed by pre-treatment with the antioxidant NAC, which significantly reduced ROS levels in both models. This reduction was particularly evident in DLD-1 cells, where ROS-positive populations decreased to 12.8% (BAK) and 41.3% (5-FU + BAK), indicating a strong dependence of the observed effects on intracellular ROS accumulation. When considered together with earlier findings, these results suggest a coherent sequence of events in which ROS overproduction acts as an initiating factor, leading to mitochondrial destabilization, activation of caspase-dependent apoptosis, and, in parallel, induction of autophagy. Importantly, all these processes are more pronounced in DLD-1 cells, highlighting their higher susceptibility to BAK-based treatment. In contrast, the weaker ROS response observed in HT-29 cells is consistent with their limited activation of downstream pathways, including apoptosis and autophagy, further supporting the notion of reduced sensitivity of this cell line to the combined treatment.

**Figure 12:**
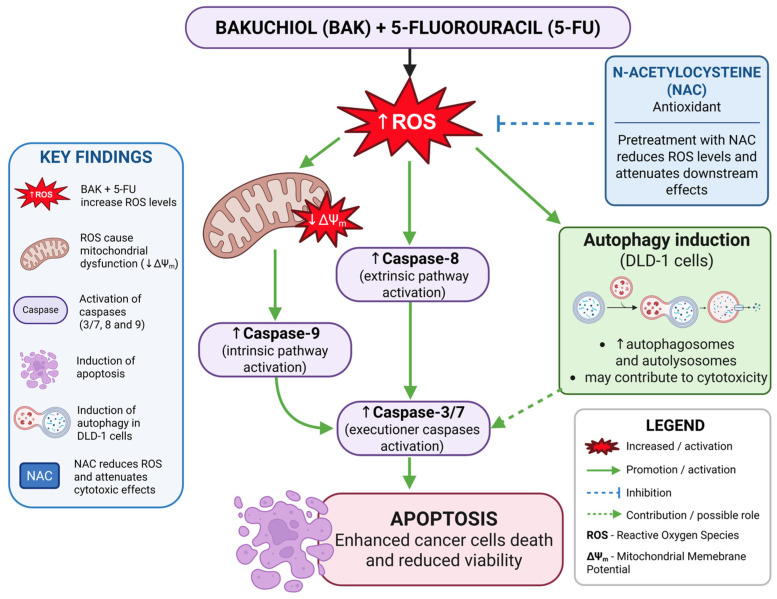
Proposed ROS-dependent mechanism of BAK and 5-FU co-treatment in colorectal cancer cells. Created in BioRender. Czarnomysy, R. (2026) https://BioRender.com/2gknhid (accessed on 17 June 2026).

Based on the obtained results, a schematic model summarizing the proposed ROS-dependent mechanism of action of the BAK and 5-FU combination is presented in Figure 12.

## 3. Discussion

Among the therapeutic strategies used in oncology, chemotherapy remains a cornerstone; however, its clinical effectiveness is increasingly limited by the development of multidrug resistance [22,23]. This challenge has driven the search for novel agents capable not only of exerting cytotoxic effects but also of sensitizing cancer cells to existing drugs. Naturally derived compounds are of particular interest in this context. In this regard, bakuchiol appears to be a promising candidate that may enhance the efficacy of standard chemotherapeutics such as 5-fluorouracil (5-FU).

The rationale for developing a combination therapy in this study was based on the assumption that BAK could potentiate the anticancer activity of 5-FU and overcome, at least partially, resistance observed in colorectal cancer models. Our results confirm this hypothesis, as co-treatment enhanced cytotoxicity and reduced the effective dose of 5-FU on the DLD-1 cell line. However, the selectivity indices obtained in this study indicate that the preferential activity toward cancer cells was moderate and depended on the cellular model. While the combination treatment generally improved selectivity compared with 5-FU alone, particularly in DLD-1 cells, lower selectivity values observed in HT-29 cells suggest that further studies are required to better characterize the balance between anticancer efficacy and potential effects on normal cells. Therefore, although the present findings support the therapeutic potential of the combination treatment in vitro, its translational relevance should be further evaluated in more advanced preclinical models.

Oxidative stress is an important regulator of cellular fate in cancer, influencing both survival and death pathways. ROS are well-established mediators of apoptosis and autophagy [19,24], and their excessive accumulation can lead to mitochondrial dysfunction and activation of cell death signaling. In the present study, BAK, particularly in combination with 5-FU, induced an increase in intracellular ROS levels, especially in DLD-1 cells. This effect was partially reversed by NAC pre-treatment, supporting the functional involvement of oxidative stress in the observed cytotoxicity. The stronger ROS accumulation in DLD-1 cells was accompanied by enhanced activation of downstream apoptotic pathways, suggesting that oxidative stress is contributing to the increased sensitivity of these cells to the combined treatment. In contrast, the weaker response observed in HT-29 cells may reflect their higher antioxidant capacity, potentially associated with efficient redox regulation mechanisms such as Nrf2 signaling [25,26]. The distinct responses observed between DLD-1 and HT-29 cells are likely related to intrinsic biological differences between these colorectal cancer models. Colorectal cancer cell lines exhibit substantial heterogeneity with respect to genetic background, metabolic activity, oxidative stress regulation, and susceptibility to apoptosis [27,28]. Previous studies have demonstrated that HT-29 cells possess a relatively efficient antioxidant defense system and a greater capacity to adapt to oxidative stress conditions, which may limit ROS accumulation and attenuate apoptosis induction. In contrast, DLD-1 cells appear to be more susceptible to treatment-induced redox imbalance, as evidenced in the present study by higher ROS levels, greater mitochondrial depolarization, stronger caspase activation, and induction of autophagy. These differences may explain the more pronounced response of DLD-1 cells to the combined BAK and 5-FU treatment and further support the role of oxidative stress as a determinant of treatment sensitivity.

The ability of BAK to disturb intracellular redox homeostasis may be particularly relevant in the context of resistance to fluoropyrimidine-based chemotherapy. Previous studies have shown that resistance to 5-FU is frequently associated with enhanced antioxidant defense systems, increased glutathione levels, activation of the Nrf2 pathway, and adaptation to oxidative stress, which collectively reduce susceptibility to chemotherapy-induced cell death [25,26]. Such adaptations enable cancer cells to survive despite ROS generation and DNA damage induced by anticancer agents. In the present study, the marked increase in ROS levels induced by BAK and the reversal of this effect by NAC suggest that oxidative stress is a key mediator of the observed biological response. Therefore, BAK may partially counteract cellular antioxidant defenses and sensitize colorectal cancer cells to 5-FU through disruption of redox homeostasis.

The reduction in cell viability observed in our study was closely associated with the induction of apoptosis, the predominant form of programmed cell death in response to anticancer treatment [20,29,30]. In line with previous studies demonstrating the proapoptotic activity of BAK [11], the combination of BAK and 5-FU significantly increased the proportion of apoptotic cells, particularly in DLD-1 cells, while maintaining relatively low levels of necrosis. This suggests that the treatment promotes a controlled and efficient mode of cell elimination. The consistency between apoptosis assays and MTT results further supports the role of BAK as a sensitizing agent that enhances the response to 5-FU.

Although BAK alone exhibited notable cytotoxic and proapoptotic activity, it cannot be considered a substitute for 5-FU, which remains a clinically established antimetabolite with a well-defined mechanism of action. Instead, BAK appears to act primarily as a modulator of cellular stress responses, including ROS generation and mitochondrial dysfunction [31]. This is supported by the observed loss of mitochondrial membrane potential (ΔΨ_m_), particularly in DLD-1 cells, accompanied by activation of caspase 9, indicating involvement of the intrinsic apoptotic pathway [18,21]. Activation of caspase 8 suggests that the extrinsic pathway may also contribute, although to a lesser extent [17,20]. The subsequent activation of executioner caspases 3 and 7 confirms effective propagation of the apoptotic signal [7,8,10,12,32], again more pronounced in DLD-1 cells. The present findings are also consistent with previous reports demonstrating that BAK induces ROS-dependent apoptosis through mitochondrial dysfunction and caspase activation in various cancer models [7,31]. The observed loss of mitochondrial membrane potential, activation of caspase 9, increased expression of caspase 3, 7, 8, and 9, and attenuation of these effects following NAC treatment collectively support a model in which ROS accumulation acts as an upstream signal triggering mitochondrial apoptosis. Therefore, redox modulation appears to be a central mechanism through which BAK enhances the biological activity of 5-FU in colorectal cancer cells.

In addition to apoptosis, autophagy was evaluated as a potential cellular response. As a context-dependent process, autophagy may either promote survival or contribute to cell death [31,32,33,34,35]. In the present study, autophagy was only induced in DLD-1 cells and further enhanced under combination treatment. While this may indicate a supportive role in cytotoxicity, its exact function remains unclear and requires further investigation using specific modulators of the autophagic pathway. The absence of a comparable response in HT-29 cells may reflect differences in their capacity to adapt to treatment-induced stress.

The results of this study suggest that BAK enhances the anticancer activity of 5-FU through a mechanism involving oxidative stress, mitochondrial dysfunction, and activation of apoptotic pathways, with a possible contribution of autophagy. The stronger effects observed in DLD-1 cells further highlight differences in cellular susceptibility, potentially related to variations in redox regulation in the tested cell [26,36]. Although additional studies are required to fully reveal the molecular targets of BAK and confirm these findings in more complex models, the present data indicate that BAK may act as a sensitizing agent in colorectal cancer cells and warrant further investigation in advanced preclinical models, including in vivo studies, to determine its therapeutic potential in combination with conventional chemotherapy.

## 4. Materials and Methods

### 4.1. Materials

5-Fluorouracil, 3-(4,5-dimethylthiazol-2-yl)-2,5-diphenyltetrazolium bromide (MTT), bakuchiol, dimethyl sulfoxide (DMSO), formaldehyde, glycine, anti-mouse HRP-linked secondary antibody, anti-rabbit HRP-linked secondary antibody and N-acetylcysteine (NAC) were from Sigma-Aldrich (St. Louis, MO, USA). Sodium chloride was a product of Avantor Performance Materials (Gliwice, Poland). Stock cultures of human colorectal adenocarcinoma (DLD-1 (CCL-221) and HT-29 (HTB-38)) and human skin fibroblasts (BJ, CRL-2522) were obtained from the American Type Culture Collection (ATCC, Manassas, VA, USA). Dulbecco’s Minimal Eagle Medium (DMEM), fetal bovine serum (FBS), phosphate-buffered saline (PBS) used in cell culture, trypsin, glutamine, penicillin, and streptomycin were from Gibco (San Diego, CA, USA), while McCoy’s 5a medium and RPMI 1640 medium were obtained from PAN Biotech (Aidenbach, Lower Bavaria, Germany) and American Type Culture Collection (ATCC, Manassas, VA, USA), respectively. FITC Annexin V Apoptosis Detection Kit II and JC-1 MitoScreen Kit were purchased from BD Pharmigen (San Diego, CA, USA). 1,4-dithiothreitol (DTT) was a product of Roche (Basel, Switzerland). Nitrocellulose membranes were purchased from BioRad Laboratories (Hercules, CA, USA). Non-fat dried milk was a product of Santa Cruz Biotechnology, Inc. (Dallas, TX, USA). Caspase-3 Rabbit mAb, Caspase-7 Mouse mAb, Caspase-8 Rabbit mAb, and Caspase-9 Mouse mAb were purchased from Cell Signaling Technology (Danvers, MA, USA). Amersham ECL Detection Reagents were purchased from Cytiva (Marlborough, MA, USA). An Autophagy Assay kit, FAM-FLICA^®^ Caspase-3/7 Assay kit, FAM-FLICA^®^ Caspase-8 Assay kit, FAM-FLICA^®^ Caspase-9 Assay kit, and Intracellular Total ROS Activity Assay were products of ImmunoChemistry Technologies (Bloomington, MN, USA).

### 4.2. Cell Culture of DLD-1, HT-29, and Fibroblast Cells

Human colorectal adenocarcinoma cell lines (DLD-1 and HT-29) and fibroblast skin cells were from the American Type Culture Collection (ATCC, Manassas, VA, USA). DLD-1 and HT-29 cells were cultured in McCoy’s 5a medium and RPMI 1640 medium, respectively, while fibroblast cells were cultured in Dulbecco’s Modified Eagle Medium (Gibco, San Diego, CA, USA). All media were supplemented by 10% of fetal bovine serum (FBS) and 1% of antibiotics: penicillin and streptomycin (both from Gibco, San Diego, CA, USA). The cells were maintained in an incubator that provides the optimal growth conditions for the cell culture: 5% CO_2_, 37 °C, and humidity in a range of 90–95%. The cells were cultured in 100 mm plates (Sarstedt, Newton, NC, USA). Subsequently, after obtaining a subconfluent cell culture, the cells were detached with 0.05% trypsin with 0.02% EDTA (Gibco, San Diego, CA, USA). Then, utilizing a Scepter 3.0 handheld automated cell counter (Milipore, Burlington, MA, USA), the number of cells was quantified and seeded at a density of 5 × 10^5^ cells per well in 24-well plates (“Nunc”) in 2 mL of the growth medium (McCoy’s 5a medium, RPMI 1640 medium, or Dulbecco’s Modified Eagle Medium, respectively). In the present study, cells that obtained 80% of confluence were used.

### 4.3. Cell Viability Assay

The cytotoxic activity of the tested compounds towards DLD-1 and HT-29 colorectal adenocarcinoma cells and human skin fibroblasts was determined by MTT assay. All cultured cell lines were treated with different concentrations of the tested compounds in medium (0.5, 1, 2.5, 7.5, 15, and 30 µg/mL) and incubated for 24 h in 96-well plates (baseline seeding density: 1 × 10^4^ cells/well). Cells were then incubated for 2 h at 37 °C with a 5 mg/mL solution of 3-(4,5-dimethyl-2-thiazolyl)-2,5-diphenyl-2H-tetrazolium bromide (Sigma-Aldrich, St. Louis, MO, USA) prepared in PBS (Corning, Kennebunk, ME, USA). Following incubation, the cells were lysed using 100 μL of lysis buffer (pH 4.7) containing 5% SDS, 3% N,N-dimethylmethanamide (DMF), 2% acetic acid, and 25 mM HCl. The absorbance was determined at a wavelength of 570 nm using an Absorbance 96 microplate reader (Byonoy GmbH, Hamburg, Germany) [37].

### 4.4. Flow Cytometry Assessment of Annexin V Binding

To determine the type of cell death induced by 5-FU and BAK and their combined therapy, analysis was performed using a flow cytometer (BD FACSCanto II, BD Biosciences Systems, San Jose, CA, USA) and Apoptosis Detection Kit II (BD Pharmingen, San Diego, CA, USA). The assay was performed according to the manufacturer’s instructions. DLD-1 and HT-29 colorectal adenocarcinoma cells were incubated for 24 h with 10 µg/mL 5-FU and BAK and as a combination of these two compounds at a concentration of 5-FU 5 µg/mL + BAK 10 µg/mL. After that cells were deprived of medium and washed several times with cold PBS. A cell suspension was prepared in the binding buffer provided in the kit at a concentration of 1 × 10^6^ cells/mL. From each sample, 100 µL of cell suspension was collected and transferred to tubes to which 5 µL of Annexin V-FITC and 5 µL of propidium iodide (PI) were added. The tubes were incubated for 15 min at room temperature in the dark. After incubation, the contents of the tubes were made up to 500 µL with a binding buffer and immediately read in a BD FACSCanto II flow cytometer (Becton Dickinson Biosciences, San Jose, CA, USA). The equipment was calibrated with BD Cytometer Setup and Tracking Beads (BD Biosciences, San Diego, CA, USA).

### 4.5. Cytometric Analysis of Mitochondrial Membrane Potential

Disruption of the mitochondrial membrane potential (MMP, ΔΨ_m_) was detected using the lipophilic cationic carbocyanine fluorochrome JC-1 (BD Pharmigen, San Diego, CA, USA). The entire assay was performed according to the manufacturer’s instructions provided with the purchased kit. DLD-1 and HT-29 cells were incubated for 24 h with 5-FU and BAK (concentrations of 10 and 5 + 10 µg/mL in monotherapy and combination therapy, respectively). After the incubation period, the cells (1 × 10^6^ cells/sample) were washed and resuspended in 0.5 mL of buffer containing 10 µg/mL JC-1 dye. Incubation was continued in the dark for 15 min at room temperature. Afterwards, the cells were washed twice with buffer, resuspended in 0.5 mL PBS, and immediately analyzed using a flow cytometer (10,000 events measured) and FACSDiva v6.1.3 software (both from BD Biosciences Systems, San Jose, CA, USA) to count the percentage of cells with reduced ΔΨ_m_. The equipment was calibrated with BD Cytometer Setup and Tracking Beads (BD Biosciences, San Diego, CA, USA).

### 4.6. Caspase 3/7, 8, and 9 Enzymatic Activity Assay

The activities of initiator caspases (caspases 8 and 9) and executor caspases (caspases 3 and 7) were assessed using FAM-FLICA^®^ Caspase Assays kits (all from ImmunoChemistry Technologies, Bloomington, MN, USA). The entire procedure was performed according to the instructions provided by the manufacturer. The cells were treated with 10 (monotherapy) and 5 + 10 µg/mL (combination therapy) of 5-FU and the tested compound (BAK) for 24 h and then collected, washed twice with cold PBS, and resuspended in Apoptosis Wash Buffer to a final concentration of 5 × 10^5^ cells/mL. In the next step, 290 µL of each cell suspension was taken and transferred into tubes. Then, 10 µL each of FLICA solution diluted immediately before use (1:5 *v*/*v*, using PBS) was added to the cells, mixed by pipetting, and incubated for 1 h at 37 °C, protected from light. After the incubation period, the cells were washed twice with 2 mL Apoptosis Wash Buffer, centrifuged, and resuspended in 300 µL of the buffer. Thus, prepared samples were immediately analyzed using a BD FACSCanto II flow cytometer (10,000 events) with FACSDiva software (both from BD Biosciences Systems, San Jose, CA, USA). The equipment calibration was performed using BD Cytometer Setup and Tracking Beads (BD Biosciences, San Diego, CA, USA).

### 4.7. Western Immunoblotting

HT-29 and DLD-1 cells were treated with 10 (monotherapy) and 5 + 10 µg/mL (combination therapy) of 5-FU and the tested compound (BAK) for 24 h and then harvested using cell lysis buffer supplemented with a protease/phosphatase inhibitor cocktail. Protein concentrations in the samples were determined using the Lowry [38]. Samples were prepared containing equal amounts of protein (30 µg/lane) and an appropriate amount of Laemmli buffer (120 mM Tris-HCl, 20% glycerol, 0.4% SDS, and 0.02% bromophenol blue, pH 6.8) containing 100 mM 1,4-dithiothreitol (DTT) (Roche, Basel, Switzerland). Samples were denatured at 95 °C for 10 min. The proteins were separated using the SDS-PAGE method described by Laemmli [39]. Proteins were separated on 12% SDS-PAGE gels. After this step, the gels were washed in cold Towbin buffer (25 mM Tris, 192 mM glycine, 20% (*v*/*v*) methanol, 0.025–0.1% SDS, pH 8.3) and were transferred to 0.2-µm nitrocellulose membranes (BioRad Laboratories, Hercules, CA, USA) using the Trans-Blot apparatus (BioRad Laboratories, Hercules, CA, USA). Transfer conditions were 250 mA at 4–8 °C overnight in freshly prepared Towbin buffer. Membranes were blocked with 5% non-fat dried milk (Santa Cruz Biotechnology, Inc., Dallas, TX, USA) in TBS-T (20 mM Tris, 150 mM NaCl, 0.1% Tween-20, pH 7.6) for 1 h at room temperature with gentle shaking. After the blocking step, membranes were washed four times with TBS-T (4 × 15 min) and incubated with primary monoclonal antibodies. Membranes were incubated with the following primary antibodies overnight at 4 °C: Caspase-3 Rabbit mAb (1:1000), Caspase-7 Mouse mAb (1:1000), Caspase-8 Rabbit mAb (1:1000), and Caspase-9 Mouse mAb (1:1000) purchased from Cell Signaling Technology (Danvers, MA, USA). Then, the membranes were again washed four times with TBS-T (4 × 15 min) and incubated with the appropriate anti-mouse or anti-rabbit HRP-linked secondary antibody (Sigma-Aldrich, St. Louis, MO, USA) at a concentration of 1:1000 in 5% non-fat milk dried in TBS-T for 1 h at room temperature with gentle shaking. Membranes were washed four times with TBS-T (4 × 15 min) and visualized using Amersham ECL Detection Reagents (Cytiva, Marlborough, MA, USA). Images were taken using BioSpectrum Imaging System UVP (Ultra-Violet Products Ltd., Cambridge, UK).

### 4.8. Cytometric Analysis of the Number of Autophagosomes and Autolysosomes Using the Autophagy Probe, Red

An autophagy assay was performed to evaluate whether the tested compounds induce the autophagy process in DLD-1 and HT-29 colorectal cancer cells. The stain included in the Autophagy Assay, Red kit (ImmunoChemistry Technologies, Bloomington, MN, USA) was an aliphatic molecule with the ability to enter the cell and fluoresce brightly upon binding to the lipid bilayer membranes of autophagosomes and autolysosomes. The staining procedure was performed according to the manufacturer’s instructions provided with the kit. DLD-1 and HT-29 colorectal cancer cells seeded at a density of 5 × 10^5^ cells/well were treated for 24 h with 5-FU and BAK at concentrations of 10 (monotherapy) and 5 + 10 µg/mL (combination therapy). After drug treatment, the unfixed cells were washed and resuspended in PBS at a concentration of 5 × 10^5^ cells/mL. Then, 490 µL each of cell suspension was taken and transferred to test tubes, and 10 µL each of Autophagy Probe, Red solution (previously diluted 1:5 in PBS) was added and incubated (30 min, 37 °C, in the dark). After the incubation period, the cells were washed and resuspended in Cellular Assay Buffer, finally adding fixative at a ratio of 1:5 (*v*/*v*). After this step, the prepared samples were immediately measured using a flow cytometer (BD FACSCanto II; 10,000 events measured), and the percentage of cells with an occurring autophagy process was calculated using FACSDiva software (both from BD Biosciences Systems, San Jose, CA, USA). The equipment calibration was performed using BD Cytometer Setup and Tracking Beads (BD Biosciences, San Diego, CA, USA).

### 4.9. Assessment of Oxidative Status Using the Intracellular Total ROS Activity Assay

The effects of 5-FU and BAK (10 µg/mL) and their combination therapy (5 + 10 µg/mL) on the induction of oxidative stress in DLD-1 and HT-29 colorectal cancer cells after 24 h exposure were evaluated by flow cytometry. The Intracellular Total ROS Activity Assay kit, which contains the Total ROS Green dye that fluoresces only after oxidation by ROS present in the cell, was used for this purpose. This assay was performed according to the manufacturer’s protocol. After 1 h pre-treatment with NAC (5 mM), followed by incubation with the tested compounds, the cells were collected, centrifuged (1200 rpm, 10 min, 4 °C), and washed twice with cold PBS solution. Then, the cells were resuspended in assay buffer attached to the kit (1:10 dilution with deionized water; cell density 1 × 10^6^ cells/mL), and Total ROS Green dye was added at a ratio of 1:50 *v*/*v*. The samples were incubated for 1 h at 37 °C in a CO_2_ incubator. After the required incubation time, the cells were washed and resuspended in 500 µL assay buffer. Thus, prepared samples were immediately analyzed by a FACSCanto II flow cytometer (10,000 measured events) with FACSDiva software (both from BD Biosciences Systems, San Jose, CA, USA). The equipment was calibrated with BD Cytometer Setup and Tracking Beads (BD Biosciences, San Diego, CA, USA).

### 4.10. Statistical Analysis

All results are presented as mean ± standard deviation (SD) from at least three independent biological experiments (*n* = 3), each performed in triplicate. Statistical analyses were performed using GraphPad Prism 8 software (GraphPad Software, San Diego, CA, USA). Comparisons between treated groups and the untreated control group were conducted using one-way analysis of variance (ANOVA) followed by Dunnett’s multiple comparison post hoc test. Differences were considered statistically significant at *p* < 0.05. Statistical significance was denoted as * *p* < 0.05, ** *p* < 0.01, and *** *p* < 0.001.

## 5. Conclusions

In conclusion, our study demonstrates that bakuchiol (BAK) enhances the anticancer activity of 5-fluorouracil (5-FU) in colorectal cancer cells, primarily through a ROS-dependent mechanism. The combined treatment promotes oxidative stress, leading to mitochondrial dysfunction, activation of both intrinsic and extrinsic apoptotic pathways, and robust executioner caspase activation. In addition, autophagy was induced in DLD-1 cells, suggesting its involvement as a complementary response to treatment-induced stress. Importantly, although BAK alone exhibited significant biological activity, its most relevant effect lies in its ability to sensitize cancer cells to 5-FU. This was reflected in the consistently stronger response observed under combination treatment, particularly in DLD-1 cells, which showed higher susceptibility across all analyzed parameters. These findings indicate that BAK may serve as a redox-modulating adjuvant capable of enhancing the effectiveness of conventional chemotherapy and potentially contributing to the reduction of drug resistance in colorectal cancer.

## Figures and Tables

**Table 1:** Selectivity and synergy indices for BAK and 5-FU combination treatment.

Cell Line	Treatment	IC_50_ (µg/mL)	SI (vs. Fibroblasts)	Combination Effect Index
DLD-1	BAK	15.40 ± 1.95	1.25	-
DLD-1	5-FU + BAK (5 µg/mL)	8.84 ± 0.15	1.22	1.74
DLD-1	5-FU + BAK (10 µg/mL)	4.52 ± 1.94	1.64	3.41
HT-29	BAK	17.78 ± 1.40	1.08	-
HT-29	5-FU + BAK (5 µg/mL)	9.38 ± 0.46	1.15	1.9
HT-29	5-FU + BAK (10 µg/mL)	9.92 ± 1.72	0.75	1.79

## Data Availability

The original contributions presented in this study are included in the article/Supplementary Materials. Further inquiries can be directed to the corresponding author.

## Supplementary Materials

The following supporting information can be downloaded at: https://www.mdpi.com/article/10.3390/ijms27135894/s1.
