# Supplementary material for: Bakuchiol Enhances 5-Fluorouracil Efficacy in Colorectal Cancer Cells via a ROS-Dependent Mechanism Involving Mitochondrial Dysfunction and Apoptosis
Source: Int J Mol Sci. 2026 Jun 30;27(13):5894. doi: 10.3390/ijms27135894 (PMC13361224; doi:10.3390/ijms27135894)
Supplement: Supplementary file 1 [file ijms-27-05894-s001.zip › ijms-4361617-supplementary.pdf]

Supplementary Materials for

Bakuchiol Enhances 5-Fluorouracil Efficacy in Colorectal Cancer Cells via a ROS-Dependent Mechanism Involving Mitochondrial Dysfunction and Apoptosis

submitted by Dominika Radomska[1], Olga Szewczyk-Roszczenko[1], Magda Chalecka[2], Arkadiusz Surazynski[2], Anna Szymanowska[1], Krzysztof Bielawski[1], and Robert Czarnomysy[1*]

[1] Department of Synthesis and Technology of Drugs, Medical University of Bialystok, Kilinskiego 1, 15-089 Bialystok, Poland
[2] Department of Medicinal Chemistry, Medical University of Bialystok, Mickiewicza 2D, 15-222 Bialystok, Poland

[*] Correspondence: e-mail: robert.czarnomysy@umb.edu.pl; Tel.: +48 85 748 57 43.

This document includes representative original membrane images from the western blot used for Figure 9.

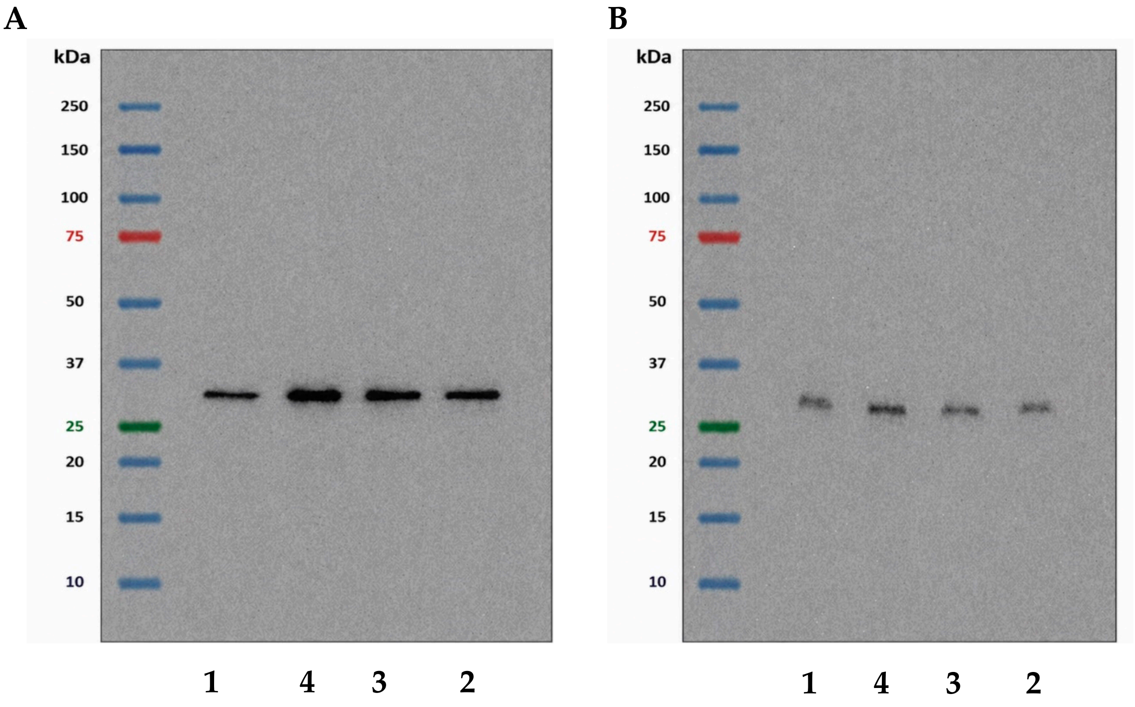

Figure S1. Original western blot membranes used to evaluate the expression of caspase 3 in DLD-1 (**A**) and HT-29 (**B**) colorectal cancer cells. 1) Control; 2) 5-FU 10 μg/mL; 3) BAK 10 μg/mL; 4) 5-FU 5 μg/mL + BAK 10 μg/mL.

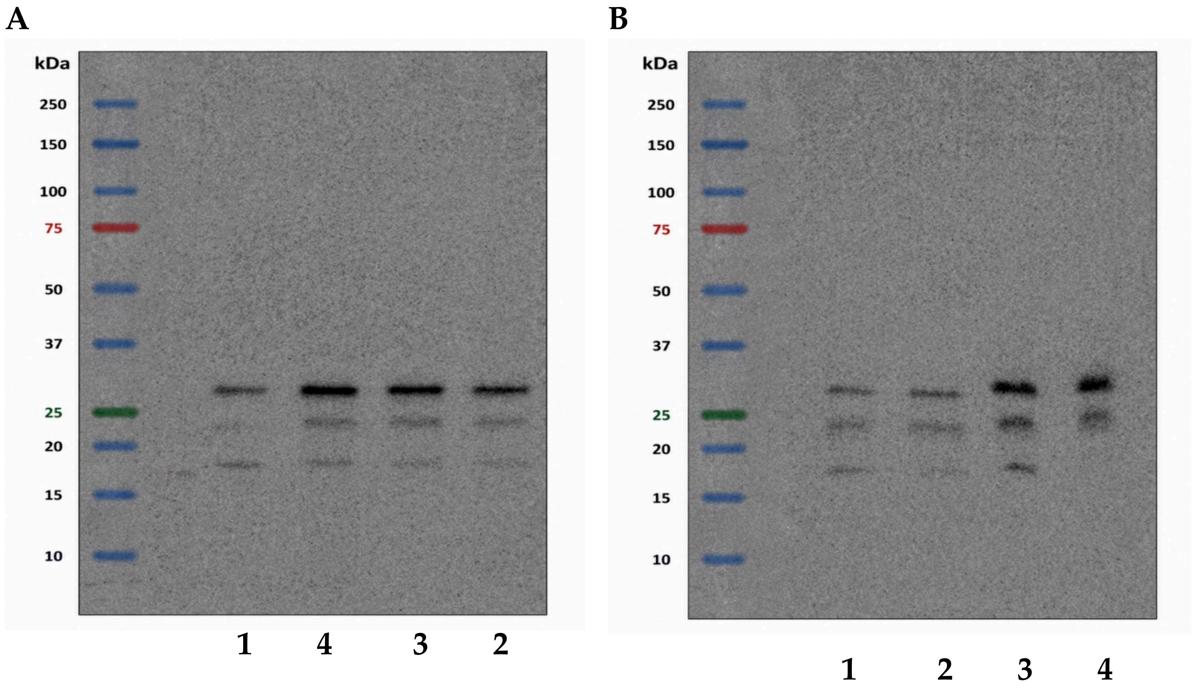

Figure S2. Original western blot membranes used to evaluate the expression of caspase 7 in DLD-1 (**A**) and HT-29 (**B**) colorectal cancer cells. 1) Control; 2) 5-FU 10 µg/mL; 3) BAK 10 µg/mL; 4) 5-FU 5 µg/mL + BAK 10 µg/mL.

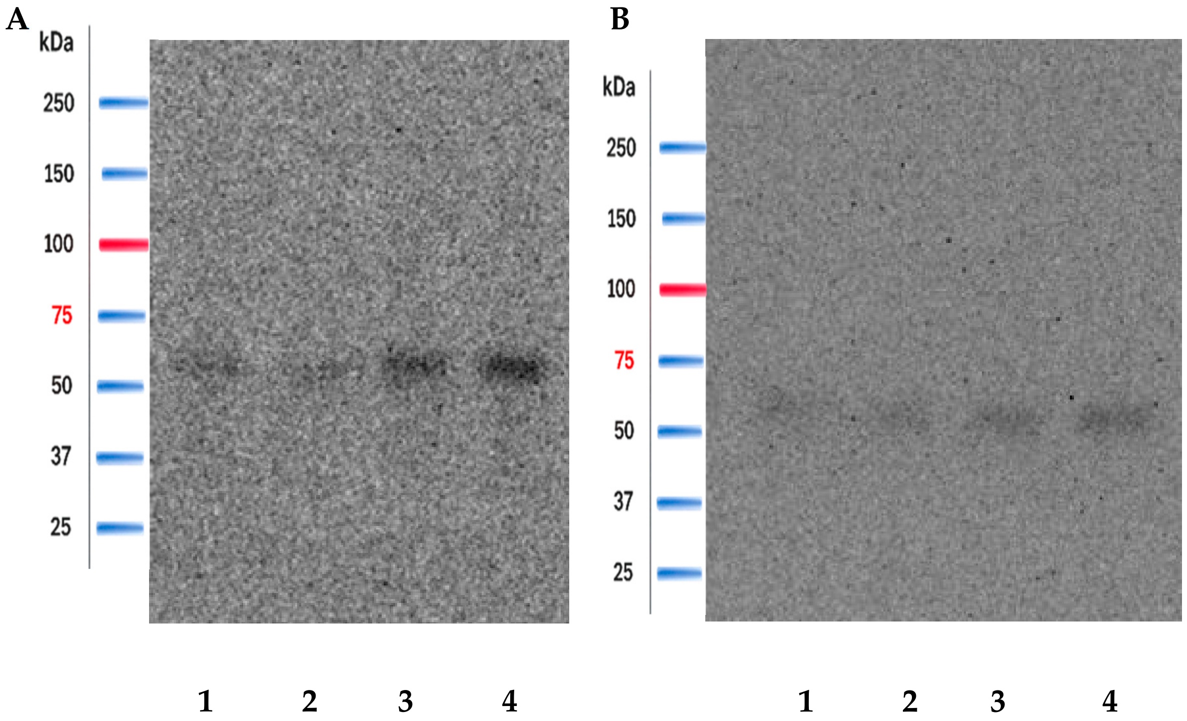

Figure S3. Original western blot membranes used to evaluate the expression of caspase 8 in DLD-1 (**A**) and HT-29 (**B**) colorectal cancer cells. 1) Control; 2) 5-FU 10 µg/mL; 3) BAK 10 µg/mL; 4) 5-FU 5 µg/mL + BAK 10 µg/mL.

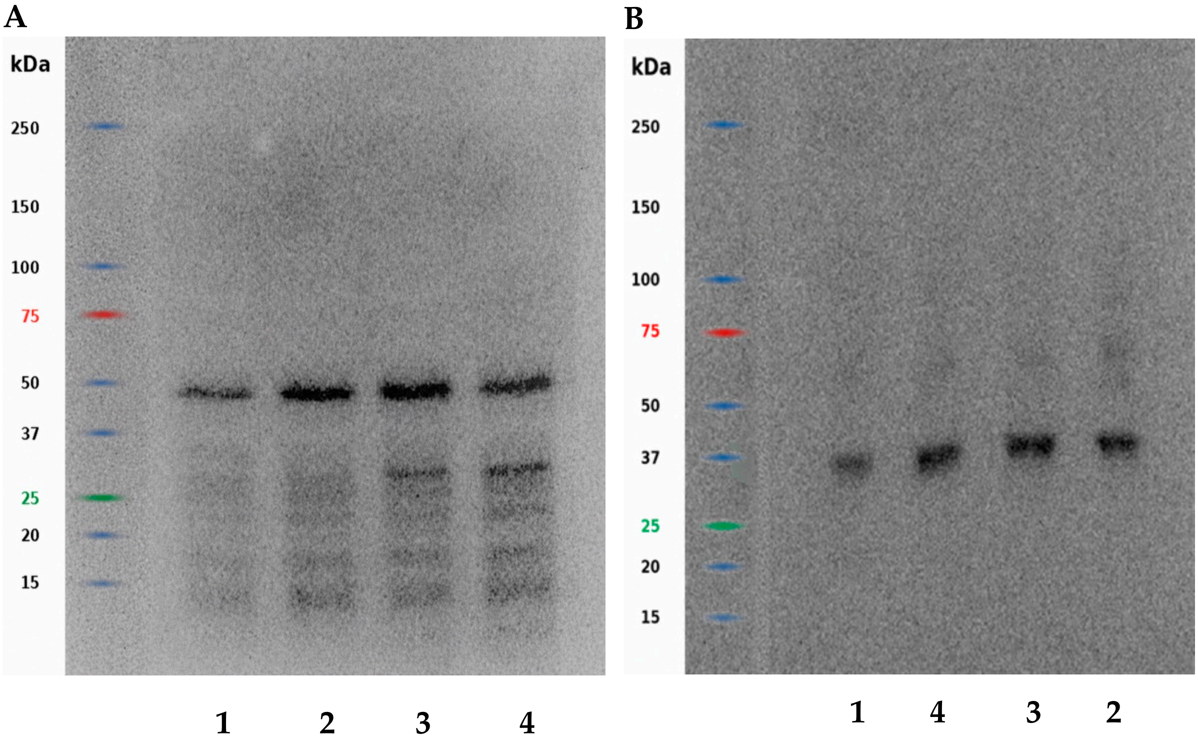

Figure S4. Original western blot membranes used to evaluate the expression of caspase 9 in DLD-1 (**A**) and HT-29 (**B**) colorectal cancer cells. 1) Control; 2) 5-FU 10 µg/mL; 3) BAK 10 µg/mL; 4) 5-FU 5 µg/mL + BAK 10 µg/mL.

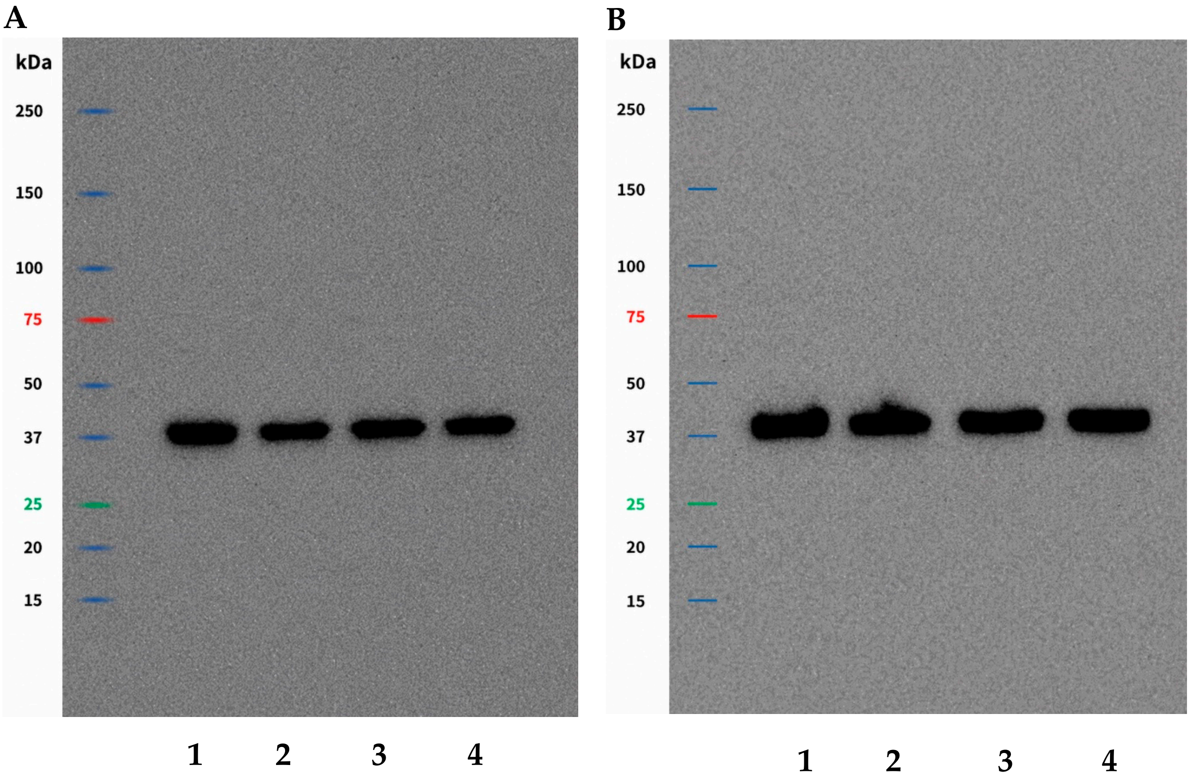

Figure S5. Original western blot membranes used to evaluate the expression of β-actin in DLD-1 (**A**) and HT-29 (**B**) colorectal cancer cells. 1) Control; 2) 5-FU 10 µg/mL; 3) BAK 10 µg/mL; 4) 5-FU 5 µg/mL + BAK 10 µg/mL.
